# Supplementary material for: Content-rich biological network constructed by mining PubMed abstracts
Source: BMC Bioinformatics. 2004 Oct 8;5:147. doi: 10.1186/1471-2105-5-147 (PMC528731; doi:10.1186/1471-2105-5-147)
Supplement: Additional File 5 — The original Chilibot query results of the term "long-term potentiation (LTP)" and 22 other terms, limiting the latest references analyzed to the years 1990, 1995, 2000, and 2004. [file 1471-2105-5-147-S5.bz2 › chilibotAdditionalFile5/ltp2000/html/PKC.html]

 


**PKC** (Input: PKC ) 

---


**Google Searches:** Entire Web  | EDU domain only  | PDF files only

.

**External Links:** OMIM |
LocusLink
| Swissprot
| GeneCards

  
**Maps of PKC**

Simple
Complete
graph in
radiant
tree
square
layout.

**New Hypothesis !**

**Synonyms** 

- pkc   [PubMed]

**Synopsis**

- Our results demonstrate that the gamma isotype of **PKC** is NOT essential for phorbol ester mediated synaptic facilitation, and place limitations on the possible roles of **PKC** in LTP.  Learn MemLearn Mem, 2000 
  [24]

- These results suggest that **PKC** may couple the postsynaptic mGluR5 to the NMDA receptor potentiation during LTP,.  Learn MemLearn Mem, 2000 
  [23]

- The results of the present study thus suggest that arachidonic acid sustains enhanced activity of nicotinic ACh receptors by interacting with a **PKC** pathway, thereby increasing glutamate release from presynaptic terminals, and then leading to an LTP like facilitation of hippocampal synaptic transmission.  Brain Res Mol Brain Res, 1999 
  [20]

- These results suggest that the increase of calcium influx through NMDA channels and L type VOCCs in turn triggering a **PKC** dependent signaling cascade is a possible cellular basis underlying this seizure like activity induced inhibition of LTP.  J Neurophysiol, 2000 
  [20]

- These results suggest that the compartmentalization of **PKC** isoforms in neurons may contribute to their function, with the location of PKMzeta prominent in areas notable for long term synaptic plasticity.  J Comp Neurol, 2000 
  [19]

- However, **PKC** is involved in the regulation of LTP induction, via the molecular switch process.  Eur J Neurosci, 2000 
  [19]

- These data show that in LTP K the in situ phosphorylation of pre and postsynaptic **PKC** substrates is increased, indicating that NMDA receptor dependent and NMDA receptor independent LTP share common calcium dependent expression mechanisms, including activation of pre and postsynaptic **PKC**.  Neurosci Lett, 2000 
  [19]

- **PKC** potentiation of NMDAR function is likely to be an important mode of NMDAR regulation in vivo and may play a role in NMDA dependent long term potentiation  [LTP] .  Proc Natl Acad Sci U S A, 1999 
  [19]

- When the NMDA channels are open, the determinant role in receptor phosphorylation is played by the **PKC** and CaMKII.  Ross Fiziol Zh Im I M Sechenova, 2000 
  [19]

- Together these findings show that activity dependent synaptic plasticity elicits **PKC** mediated phosphorylation of substrate proteins in a highly selective and coordinated manner and demonstrate the compartmentalization of **PKC** substrate interactions.  J Neurochem, 1999 
  [16]

- The **PKC** expression pattern and behavioral phenotype in the **PKC** beta knock out animals indicate a critical role for the beta isoform of **PKC** in learning related signal transduction mechanisms, potentially in the basolateral nucleus of the amygdala.  J Neurosci, 2000 
  [16]

- It is suggested that the induction of LTD by LFS involves activation of **PKC** and PKA following activation of group I and group II metabotropic glutamate receptors mGluR .  Neurosci Lett, 1999 
  [16]

- Thus, the present findings demonstrate the participation of PKCbetaI in the early synaptic events responsible for the acquisition and consolidation of an inhibitory avoidance learning, and suggest a putative role of this presynaptic isozyme on the enhanced **PKC** dependent B 50 GAP 43 phosphorylation previously detected by us during this associative learning.  Brain Res, 2000 
  [16]

- Our results are consistent with the hypothesis that the effects of lead on LTP are mediated via an action on **PKC**, which varies with age.  Brain Res Dev Brain Res, 2000 
  [14]

- These results indicate that postsynaptic CaM KII and **PKC** activities are essential for maintaining LTP expression, but CaN activity limits synaptic strength at stable levels during both basal and potentiated synaptic transmission.  Learn MemLearn Mem, 2000 
  [13]
